# An Improved Adaptive Received Beamforming for Nested Frequency Offset and Nested Array FDA-MIMO Radar

**DOI:** 10.3390/s18020520

**Published:** 2018-02-08

**Authors:** Sibei Cheng, Qingjun Zhang, Mingming Bian, Xinhong Hao

**Affiliations:** 1 National Key Laboratory of Mechatronic Engineering and Control, Beijing Institute of Technology, Beijing 100081, China; sibeicheng@bit.edu.cn (S.C.); haoxinhong@bit.edu.cn (X.H.); 2 Beijing Institute of Spacecraft System Engineering, Beijing 100086, China; bianmingming2008@163.com

**Keywords:** FDA-MIMO, nested frequency offset, nested array, adaptive beamforming

## Abstract

For the conventional FDA-MIMO (frequency diversity array multiple-input-multiple-output) Radar with uniform frequency offset and uniform linear array, the DOFs (degrees of freedom) of the adaptive beamformer are limited by the number of elements. A better performance—for example, a better suppression for strong interferences and a more desirable trade-off between the main lobe and side lobe—can be achieved with a greater number of DOFs. In order to obtain larger DOFs, this paper researches the signal model of the FDA-MIMO Radar with nested frequency offset and nested array, then proposes an improved adaptive beamforming method that uses the augmented matrix instead of the covariance matrix to calculate the optimum weight vectors and can be used to improve the output performances of FDA-MIMO Radar with the same element number or reduce the element number while maintain the approximate output performances such as the received beampattern, the main lobe width, side lobe depths and the output SINR (signal-to-interference-noise ratio). The effectiveness of the proposed scheme is verified by simulations.

## 1. Introduction

As a new kind of MIMO Radar, the FDA-MIMO Radar has drawn much attention, and has been widely investigated since the introduction of the concept of the “frequency diversity array”. In contrast to the traditional MIMO Radar, the FDA-MIMO Radar consists of a beampattern that expands from the angle domain to the joint angle-range domain by utilizing a small frequency offset across the array [1]. Previous research has shown that the beampattern periodically changes with the following parameters: angle, range and time [2,3,4]. Simulation results for transmitted beampattern have been achieved by evaluating the effect of frequency offset, range, angle, antenna element spacing, as well as other factors [5],with the authors proposing that “FDA-MIMO Developments: Windowing and Nonlinear Frequency Shift”. Furthermore, in [6,7], the authors investigated the FDA-MIMO Radar with jamming signals, and established mathematical models for the FDA-MIMO Radar received signals and its jamming signals.

In recent years, the FDA-MIMO Radar has continued toplace a lot of emphasis on the exploitation of the benefits that are exclusive to the joint angle-range domain. In particular, range-angle location and estimation has been a popular research subject [7,8,9,10]. For instance, a joint range-angle estimation algorithm is presented in [7]; a FDA-MIMO Radar with double pulsewas proposed, with the aim ofimproving the range-angle localization of the target [8]. Another popular area of FDA-MIMO Radar research is related to optimizing nonuniform frequency offset to obtain modified range-angle beampattern or better output performance [11,12,13,14,15]. As discussed in [11], the logarithmic frequency offset allows a single maxima for each beam, ensuring that the signal information at the receiver is of better quality. Moreover, square increasing and cubic frequency offset are recommended for the FDA-MIMO Radar, so that the targets’ range and angle can be estimated without ambiguities [12]. A cognitive FDA-MIMO Radar with situational awareness is researched to maximize the output SINR by iteratively optimizing the frequency offset in a closed-loop control manner [13]. In [15], the authors took advantage of an optimal frequency increment selection method by maximizing the SINR in each coherent processing interval for the FDA-MIMO Radar, and also discuss a corresponding target discrimination method. Nevertheless, to the best of our knowledge, less attention has been paid to tapping the potential of nonuniform frequency offset in increasing the DOFs while keeping the same element number.

Larger DOFs in the angle domain have been achieved by choosing a suitable nonuniform array (or sparse array), such as MRA (minimum redundancy array) [16], co-prime array [17], and nested array [18]. As mentioned above, the beampattern of FDA-MIMO Radar, which involves the joint angle-range domain and the corresponding DOFs, is decided by both the frequency offset and the array interval. Consequently, increasing the DOFs in the FDA-MIMO Radar has to be achieved based on these two aspects. We research the signal model of the FDA-MIMO Radar with nested frequency offset and nested array, and propose an improved MVDR (Minimum Variance Distortionless Response) beamforming. Rather than directly using the covariance matrix obtained from the received data matrix, the improved MVDR beamforming method augments the covariance matrix of the received data with a new Toeplitz matrix that can provide greater DOFs. By using the new matrix in the MVDR beamforming, the resultant beampattern has lower side lobes and higher SINR than the conventional MVDR beamforming, which utilizes the covariance matrix in a direct manner.

The rest of the paper is organized as follows: Signal models of the collocated FDA-MIMO Radar with nested frequency offset and nested array are presented in Section 2. This Section explains the specific process of the improved MVDR beamforming. Simulations and discussions are shown in Section 3, followed by the conclusions in Section 4.

## 2. Signal Models of the Collocated NNFDA-MIMO Radar

An *N*-element collocated FDA-MIMO Radar with the nested array and the nested frequency offset, namedan NNFDA-MIMO Radar, transmits signals and receives the echo signals using the same two-level nested array. A two-level nested array is basically a concatenation of two ULAs (uniform linear arrays): inner ULA and outer ULA, where the inner ULA has $N_{1}$ elements with interval d and the outer ULA has $N_{2}$ elements with interval $(N_{1}+1)d$. The array intervalof each omnidirectional element is half wavelength, expressed as $d=\frac{\lambda }{2}=\frac{c}{2f_{0}}$, c is the light speed. When N is even, $N_{1}=N_{2}=\frac{N}{2}$, while when N is odd, $N_{1}=\frac{N-1}{2}$ and $N_{2}=\frac{N+1}{2}$. Consequently, for the *N*-element two-level nested array, the position set is $P=[d,2d,\ldots ,N_{1}d,(N_{1}+1)d,2(N_{1}+1)d,\ldots ,N_{2}(N_{1}+1)d]$.

**Figure 1:**
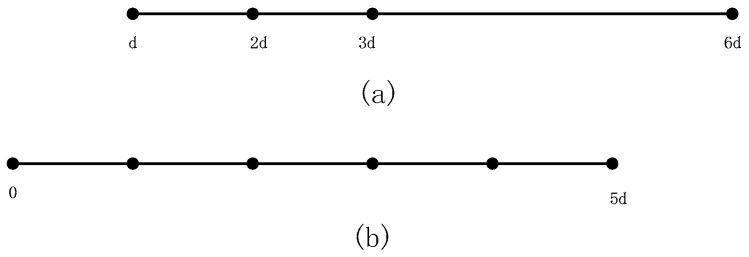
(**a**) The 4-element two-level nested array; (**b**) The 6-element uniform linear array.

To illustrate the difference between the two-level nested array and ULA, we show sketches of the 4-element two-level nested array and the 6-element ULA in Figure 1a,b.

**Figure 2:**
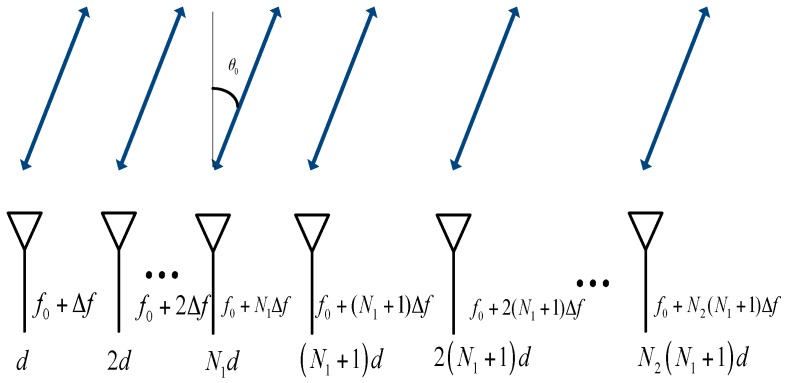
The transmit and receive scheme of NNFDA-MIMO Radar.

The *n*th transmit element’s carrier frequency $f_{n}$ is expressed as

(1) $$f_{n}=f_{0}+J_{n}\Delta f$$

where $f_{0}$ is the reference carrier frequency and Δf is the frequency offset, which is negligible compared with $f_{0}$. $J_{n}$ is the *n*th element of the set $J=[1,2,\ldots ,N_{1},(N_{1}+1),2(N_{1}+1),\ldots ,N_{2}(N_{1}+1)]$, $N_{1}=N_{2}=\frac{N}{2}$ when N is even, while $N_{1}=\frac{N-1}{2}$ and $N_{2}=\frac{N+1}{2}$ when N is odd. The *N*-element collocated NNFDA-MIMO Radar is shown schematically in Figure 2.

Given the far-field point target at range $r_{0}$ and from angle $\theta_{0}$, the signal $y_{m,n}$,which is transmitted by the *n*th element, reflected by the target, then received by the *m*th element and finally matched-filtered, can be written as

(2) $$y_{m,n}\approx \xi \exp\{-j4\pi \frac{\Delta f}{c}J_{n}r_{0}\}\times \exp\{j2\pi \frac{d}{\lambda }J_{n}\sin(\theta_{0})\}\times \exp\{j2\pi \frac{d}{\lambda }J_{m}\sin(\theta_{0})\}$$

where $\xi =\rho \exp\{j2\pi f_{0}r_{0}\}$ and ρ is the complex-valued coefficient of the point target. The received snapshot of target in FDA-MIMO can be expressed in the vector form as

(3) $$\begin{array}{cc} x_{s} & ={\left[y_{11},y_{12},\ldots ,y_{1N},y_{21},\ldots ,y_{NN}\right]}^{T}\\ & =\xi b(\theta_{0})⊗a(r_{0},\theta_{0})\\ & =\xi v_{1}(r_{0},\theta_{0}) \end{array}$$

where “⊗” denotes the Kronecker product operator and the superscript *^T^* is the transpose operator. $v_{1}(r_{0},\theta_{0})$ is the virtual steering vector, $a(r_{0},\theta_{0})$ and $b(\theta_{0})$ are the transmit steering vector and receive steering vector respectively, expressed as

(4) $$\begin{array}{l} a(r_{0},\theta_{0})={\left[e^{J_{1}\varphi (r_{0},\theta_{0})},e^{J_{2}\varphi (r_{0},\theta_{0})},\ldots ,e^{J_{N}\varphi (r_{0},\theta_{0})}\right]}^{T}\\ \varphi (r_{0},\theta_{0})=\left(-j4\pi \frac{\Delta f}{c}r_{0}+j2\pi \frac{d}{\lambda }\sin(\theta_{0})\right) \end{array}$$

(5) $$\begin{array}{l} b(\theta_{0})={\left[e^{J_{1}\phi (\theta_{0})},e^{J_{2}\phi (\theta_{0})},\ldots ,e^{J_{N}\phi (\theta_{0})}\right]}^{T}\\ \phi (\theta_{0})=\{j2\pi \frac{d}{\lambda }\sin(\theta_{0})\} \end{array}$$

The received snapshot x has the following components: the target component $x_{s}$, the interference component $x_{i}$ and the noise component $w_{n}$. Assume that there are L interferences impinging on the array from the direction $\theta_{l},l=1,2,\ldots ,L$. The received interference component can be expressed as [7]

(6) $$x_{i}=\sum_{l=1}^{L}\xi_{l}b(\theta_{l})⊗n_{al}$$

where $\xi_{l}$ is a zero-mean circularly symmetric complex Gaussian random variable with variance ${\sigma_{l}}^{2}=E\{\xi_{l}{\xi_{l}}^{H}\},l=1,2,\ldots ,L$, the superscript *^H^* is the conjunctive transpose operator. The $n_{al}\in C^{N\times 1}$ is the noise-like transmit steering vector of the noise jamming and assumed zero-mean white Gaussian distribution [19]. The $b(\theta_{l})\in C^{N\times 1}$ is the receive steering vector of the noise jamming, and takes the form $b(\theta_{l})={\left[e^{J_{1}\phi (\theta_{l})},e^{J_{2}\phi (\theta_{l})},\ldots ,e^{J_{N}\phi (\theta_{l})}\right]}^{T}$.

Hence, the total received snapshot can be written as

(7) $$\begin{array}{cc} x & =x_{S}+x_{i}+w_{n}\\ & =\xi v_{1}(r_{0},\theta_{0})+\sum_{l=1}^{L}\xi_{l}b(\theta_{l})⊗n_{al}+w_{n} \end{array}$$

There is an assumption that the noise is temporally-spatially white, and temporally uncorrelated from each signal. Additionally, the interferences are statistically noise-like and oppressive. The matrix $R_{j}=\sum_{l=1}^{L}{\sigma_{l}}^{2}\left(b(\theta_{l})b{\left(\theta_{l}\right)}^{H}\right)⊗I_{N}$ is defined as the interferences’ covariance matrix, $I_{N}$ is the N×N identity matrix. For the *N*-element collocated NNFDA-MIMO Radar, the covariance matrix R can be expressed as

(8) $$R={\sigma_{1}}^{2}v_{1}(r_{0},\theta_{0})v_{1}{\left(r_{0},\theta_{0}\right)}^{H}+R_{j}+{\sigma_{n}}^{2}I_{N^{2}}$$

where $\sigma_{1}$ is the desired signal’s power, $I_{N^{2}}$ is the $N^{2}\times N^{2}$ identity matrix, $\sigma_{n}$ is the noise power and $\sigma_{l}$ is the interference signal’s power.

For matrices $A\in C^{m\times n}$, $B\in C^{n\times k}$, $C\in C^{l\times p}$, $D\in C^{p\times q}$, (AB)⊗(CD)=(A⊗C)(B⊗D) and ${\left(A⊗B\right)}^{H}=A^{H}⊗B^{H}$. Hence, it can be obtained

(9) $$\begin{array}{cc} \left(b(\theta_{0})b{\left(\theta_{0}\right)}^{H})⊗(a(r_{0},\theta_{0})a{\left(r_{0},\theta_{0}\right)}^{H}\right) & =\left(b(\theta_{0})⊗a(r_{0},\theta_{0})\right)\left(b{\left(\theta_{0}\right)}^{H}⊗a{\left(r_{0},\theta_{0}\right)}^{H}\right)\\ & =\left(b(\theta_{0})⊗a(r_{0},\theta_{0})\right){\left(b(\theta_{0})⊗a(r_{0},\theta_{0})\right)}^{H}\\ & =v_{1}(r_{0},\theta_{0})v_{1}{\left(r_{0},\theta_{0}\right)}^{H} \end{array}$$

The covariance matrix R can further be formed as

(10) $$\begin{array}{cc} R & ={\sigma_{1}}^{2}v_{1}(r_{0},\theta_{0})v_{1}{\left(r_{0},\theta_{0}\right)}^{H}+R_{j}+{\sigma_{n}}^{2}R\\ & ={\sigma_{1}}^{2}(b(\theta_{0})b{\left(\theta_{0}\right)}^{H})⊗(a(r_{0},\theta_{0})a{\left(r_{0},\theta_{0}\right)}^{H})\\ & \;+\sum_{l=1}^{L}{\sigma_{l}}^{2}\left(b(\theta_{l})b{\left(\theta_{l}\right)}^{H}\right)⊗I_{N}+{\sigma_{n}}^{2}I_{N^{2}} \end{array}$$

Now, we define $B(\theta_{0})=b(\theta_{0})b{\left(\theta_{0}\right)}^{H}$, $A(r_{0},\theta_{0})=a(r_{0},\theta_{0})a{\left(r_{0},\theta_{0}\right)}^{H}$ and $B(\theta_{l})=b(\theta_{l})b{\left(\theta_{l}\right)}^{H}$. There is

(11) $$R={\sigma_{1}}^{2}B(\theta_{0})⊗A(r_{0},\theta_{0})+\sum_{l=1}^{L}{\sigma_{l}}^{2}B(\theta_{l})⊗I_{N}+{\sigma_{n}}^{2}I_{N^{2}}$$

Each element of matrix $B(\theta_{0})$, $B(\theta_{l})$ and $A(r_{0},\theta_{0})$ can be formed as

(12) $${\left[B(\theta_{0})\right]}_{j,k}=e^{J_{j}\phi (\theta_{0})-J_{k}\phi (\theta_{0})}$$

(13) $${\left[B(\theta_{l})\right]}_{j,k}=e^{J_{j}\phi (\theta_{l})-J_{k}\phi (\theta_{l})}$$

(14) $${\left[A(r_{0},\theta_{0})\right]}_{m,n}=e^{J_{m}\varphi (r_{0},\theta_{0})-J_{n}\varphi (r_{0},\theta_{0})}$$

where ${\left[A(r_{0},\theta_{0})\right]}_{m,n}$ ({m,n}=1,2,…,N) is the {m,n}th element of $A(r_{0},\theta_{0})$, ${\left[B(\theta_{0})\right]}_{j,k}\left(\left\{j,k\right\}=1,2,\ldots ,N\right)$ and ${\left[B(\theta_{l})\right]}_{j,k}\left(\left\{j,k\right\}=1,2,\ldots ,N\right)$ represent the {j,k}th element of $B(\theta_{0})$ and $B(\theta_{l})$, respectively. The function mod(x,y) returns the modulus after division of x by y, while the function fix(x) returns the first integer $x_{1}$ when $x_{1}\leq x$. According to the definition of the Kronecker product, ${\left[R\right]}_{m,n}\left(\left\{m,n\right\}=1,2,\ldots ,N^{2}\right)$ is the {m,n}th element of the matrix R($N^{2}\times N^{2}$ size) and can be decomposed as

(15) $$\begin{array}{cc} {\left[R\right]}_{m,n} & ={\sigma_{1}}^{2}{\left[B(\theta_{0})\right]}_{fix(\frac{m-1}{N^{2}})+1,fix(\frac{n-1}{N^{2}})+1}{\left[A(r_{0},\theta_{0})\right]}_{\mathrm{mod}(m-1,N^{2})+1,\mathrm{mod}(n-1,N^{2})+1}\\ & +\sum_{l=1}^{L}{\sigma_{l}}^{2}B{\left(\theta_{l}\right)}_{fix(\frac{m-1}{N^{2}})+1,fix(\frac{n-1}{N^{2}})+1}\delta \left(\mathrm{mod}(m-1,N^{2})-\mathrm{mod}(n-1,N^{2})\right)+{\sigma_{n}}^{2}\delta (m-n) \end{array}$$

where δ(t) is the delta function that equals 1 when t=0 and 0 when t≠0.

Here are two definitions:

(16) $$V_{1}(m,n)=J_{\left(fix(\frac{m-1}{N^{2}})+1\right)}-J_{\left(fix(\frac{n-1}{N^{2}})+1\right)}$$

(17) $$V_{2}(m,n)=J_{\left(\mathrm{mod}(m-1,N^{2})+1\right)}-J_{\left(\mathrm{mod}(n-1,N^{2})+1\right)}$$

Hence, ${\left[R\right]}_{m,n}$ is simplified as

(18) $${\left[R\right]}_{m,n}={\sigma_{1}}^{2}e^{V_{1}(m,n)\phi (\theta_{0})}\times e^{V_{2}(m,n)\varphi (r_{0},\theta_{0})}+\sum_{l=1}^{L}{\sigma_{l}}^{2}e^{V_{1}(m,n)\phi (\theta_{l})}\delta \left(V_{2}(m,n)\right)+{\sigma_{n}}^{2}\delta \left(m-n\right)$$

The principle of MVDR beamforming is minimizing the output’s variance while constraining the response of the desired signal to unity [18]. By using the augmented matrix $\bar{R}$ instead of R directly obtained from the received snapshot, we obtain a new MVDR beamformer with an optimal weight vector. It can be easily seen that the elements in R depend on the values of $V_{1}(m,n)$ and $V_{2}(m,n)$. The value of $V_{1}(m,n)$ is only decided by the array interval, while the value of $V_{2}(m,n)$ is dependent on both the frequency offset and the array interval. For the *N*-element collocated NNFDA-MIMO Radar, $V_{1}(m,n)$ and $V_{2}(m,n)$ are both elements of the following vector $V=[-(N_{2}(N_{1}+1)-1),\ldots ,-1,0,1,\ldots ,N_{2}(N_{1}+1)-1]$.

(19) $$\begin{array}{cc} D_{u} & =\sigma^{2}[e^{-J_{N_{2}(N_{1}+1)}\phi (\theta_{0})},\ldots ,1,e^{J_{1}\phi (\theta_{0})},\ldots ,e^{J_{N_{2}(N_{1}+1)}\phi (\theta_{0})}]⊗[e^{-J_{N_{2}(N1+1)}\varphi (r_{0},\theta_{0})},\ldots ,1,e^{J_{1}\varphi (r_{0},\theta_{0})},\ldots ,e^{J_{N_{2}(N_{1}+1)}\varphi (r_{0},\theta_{0})}]\\ & +\sum_{l=1}^{L}{\sigma_{l}}^{2}[e^{-J_{N_{2}(N_{1}+1)}\phi (\theta_{l})},\ldots ,e^{-J_{1}\phi (\theta_{l})},1,e^{J_{1}\phi (\theta_{l})},\ldots ,e^{J_{N_{2}(N_{1}+1)}\phi (\theta_{l})}]⊗C+{\sigma_{n}}^{2}C⊗C \end{array}$$

where $C=\left[0,0,\ldots ,0,1,0,\ldots ,0,0\right]\in C^{1\times (2N_{2}(N_{1}+1)-1)}$.

The size of $D_{u}$ is $1\times {\left(2N_{2}(N_{1}+1)-1\right)}^{2}$, so for the *N*-element collocated NNFDA-MIMO Radar, the covariance matrix R has ${\left(2N_{2}(N_{1}+1)-1\right)}^{2}$ distinct elements, and its size is $N^{2}\times N^{2}$, which means the DOFs of the adaptive beamformer is equal to $N^{2}$ [20]. Furthermore, the elements’ order of arrangement in the matrix R is given in (18), since there are ${\left(2N_{2}(N_{1}+1)-1\right)}^{2}$ distinct elements in matrix R, while the number of DOFs is just $N^{2}$. Next, we decide to rearrange the distinct elements in matrix $\bar{R}$ in a new order to augment the size of matrix R and obtain more DOFs, which could improve the output performances of the beampattern.

Firstly, we define $\bar{N}=N_{2}(N_{1}+1)$, ${\bar{J}}_{n}$ is the *n*th element of the set $\bar{J}=[0,1,\ldots ,\bar{N}-2,\bar{N}-1]\in C^{1\times \bar{N}}$ and a Toeplitz matrix $\bar{R}\in C^{{\bar{N}}^{2}\times {\bar{N}}^{2}}$, and it has $2{\bar{N}}^{2}-1$ distinct elements and can be decomposed in the following Kronecker product operator forms

(20) $$\bar{R}={\bar{R}}_{1}+{\bar{R}}_{12}⊗D_{\bar{N}}$$

(21) $$\bar{R}={\bar{R}}_{11}⊗{\bar{R}}_{12}$$

where ${\bar{R}}_{1}\in C^{{\bar{N}}^{2}\times {\bar{N}}^{2}}$, ${\bar{R}}_{11}\in C^{\bar{N}\times \bar{N}}$, ${\bar{R}}_{12}\in C^{\bar{N}\times \bar{N}}$, ${\bar{R}}_{22}\in C^{\bar{N}\times \bar{N}}$ are Toeplitz matrices and $D_{\bar{N}}$ is a $\bar{N}\times \bar{N}$ diagonal matrix. Combing the property “a linear combination of the Toeplitz matrices is still a Toeplitz matrix”, the desired Toeplitz matrix, reconstructed by the set $D_{u}$, can be written as

(22) $$\bar{R}={\sigma_{0}}^{2}\bar{B}(\theta_{0})⊗\bar{A}(r_{0},\theta_{0})+\sum_{l=1}^{L}{\sigma_{l}}^{2}\bar{B}(\theta_{l})⊗I_{\bar{N}}+{\sigma_{n}}^{2}I_{{\bar{N}}^{2}}$$

where $I_{{\bar{N}}^{2}}$ is the ${\bar{N}}^{2}\times {\bar{N}}^{2}$ identity matrix while $I_{{\bar{N}}^{2}}$ is the $\bar{N}\times \bar{N}$ identity matrix, the Toeplitz matrix $\bar{B}(\theta_{l})=\bar{b}(\theta_{l}){\left(\bar{b}(\theta_{l})\right)}^{H}$, $\bar{b}(\theta_{l})={\left[e^{{\bar{J}}_{1}\phi (\theta_{l})},e^{{\bar{J}}_{2}\phi (\theta_{l})},\ldots ,e^{{\bar{J}}_{\bar{N}}\phi (\theta_{l})}\right]}^{T}$, the Toeplitz matrix $\bar{B}(\theta_{0})=\bar{b}(\theta_{0}){\left(\bar{b}(\theta_{0})\right)}^{H}$, $\bar{b}(\theta_{0})={\left[e^{{\bar{J}}_{1}\phi (\theta_{0})},e^{{\bar{J}}_{2}\phi (\theta_{0})},\ldots ,e^{{\bar{J}}_{\bar{N}}\phi (\theta_{0})}\right]}^{T}$ and the Toeplitz matrix $\bar{A}(r_{0},\theta_{0})=\bar{a}(r_{0},\theta_{0})\bar{a}{\left(r_{0},\theta_{0}\right)}^{H}$, $\bar{a}(r_{0},\theta_{0})={\left[e^{{\bar{J}}_{1}\varphi (r_{0},\theta_{0})},e^{{\bar{J}}_{2}\varphi (r_{0},\theta_{0})},\ldots ,e^{{\bar{J}}_{\bar{N}}\varphi (r_{0},\theta_{0})}\right]}^{T}$. Define the new virtual steering vector ${\bar{v}}_{1}(r_{0},\theta_{0})=\bar{b}(\theta_{0})⊗\bar{a}(r_{0},\theta_{0})$. ${\left[\bar{R}\right]}_{m,n},\left(\left\{m,n\right\}=1,2,\ldots ,{\bar{N}}^{2}\right)$ is the {m,n}th element of the matrix $\bar{R}$, written as

(23) $${\left[\bar{R}\right]}_{m,n}={\sigma_{1}}^{2}e^{{\bar{V}}_{1}(m,n)\phi (\theta_{0})}\times e^{{\bar{V}}_{2}(m,n)\varphi (r_{0},\theta_{0})}+\sum_{l=1}^{L}{\sigma_{l}}^{2}e^{{\bar{V}}_{1}(m,n)\phi (\theta_{l})}\delta \left({\bar{V}}_{2}(m,n)\right)+{\sigma_{n}}^{2}\delta \left(m-n\right)$$

(24) $${\bar{V}}_{1}(m,n)={\bar{J}}_{\left(fix(\frac{m-1}{{\bar{N}}^{2}})+1\right)}-{\bar{J}}_{\left(fix(\frac{n-1}{{\bar{N}}^{2}})+1\right)}$$

(25) $${\bar{V}}_{2}(m,n)={\bar{J}}_{\left(\mathrm{mod}(m-1,{\bar{N}}^{2})+1\right)}-{\bar{J}}_{\left(\mathrm{mod}(n-1,{\bar{N}}^{2})+1\right)}$$

**Figure 3:**
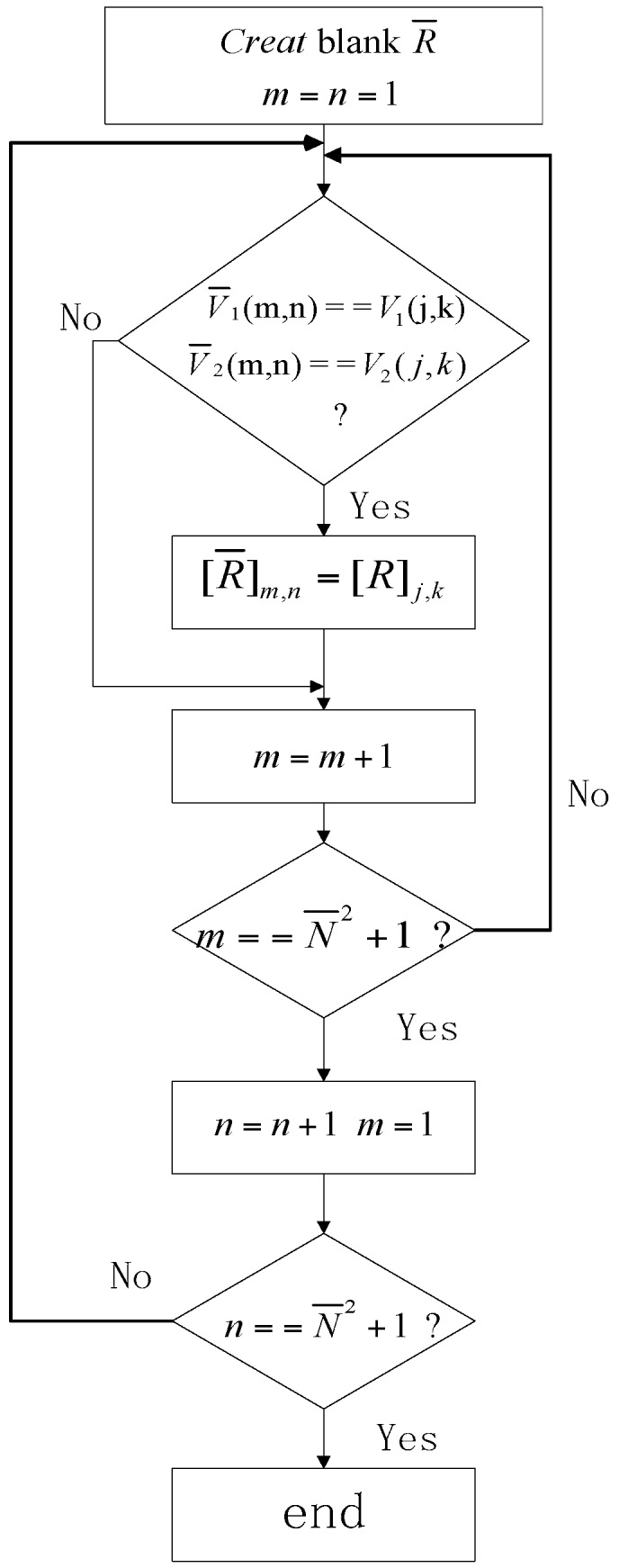
The diagram of the augmenting process.

In order to reconstruct the matrix $\bar{R}$, we need to augment the size of matrix R, so we name the Toeplitz matrix $\bar{R}$ as the corresponding augmented matrix R and the augmenting process is carried out as follows:

Step (1): Create a blank matrix $\bar{R}\in C^{{\bar{N}}^{2}\times {\bar{N}}^{2}}$ and assign the initial values m=n=1;

Step (2): Search the element ${\left[R\right]}_{j,k}$ in the matrix R ($N^{2}\times N^{2}$ size), if $V_{1}(j,k)={\bar{V}}_{1}(m,n)$ and $V_{2}(j,k)={\bar{V}}_{2}(m,n)$, assign the value of ${\left[R\right]}_{j,k}={\left[\bar{R}\right]}_{m,n}$;

Step (3): Judge the value of *m*: if $m={\bar{N}}^{2}+1$, assign m=1 and let n=n+1, otherwise let m=m+1;

Step (4): Judge the value of *n*: if $n={\bar{N}}^{2}+1$, finish the augmenting process and obtain the desired matrix $\bar{R}$ described above, otherwise repeat the Step (2);

The diagram is shown as Figure 3.

(26) $$\bar{w}={\left(\bar{R}\right)}^{-1}{\bar{v}}_{1}(r_{0},\theta_{0})$$

where ${\bar{v}}_{1}(r_{0},\theta_{0})$ is the new desired virtual steering vector, defined above and formed as

(27) $${\bar{v}}_{1}(r,\theta )={\left[e^{{\bar{J}}_{1}\phi (\theta )},e^{{\bar{J}}_{2}\phi (\theta )},\ldots ,e^{{\bar{J}}_{\bar{N}}\phi (\theta )}\right]}^{T}⊗{\left[e^{{\bar{J}}_{1}\varphi (r,\theta )},e^{{\bar{J}}_{2}\varphi (r,\theta )},\ldots ,e^{{\bar{J}}_{\bar{N}}\varphi (r,\theta )}\right]}^{T}$$

Since the weight vector $\bar{w}$ has a dimension ${\bar{N}}^{2}\times 1$, the DOFs number of the adaptive beamformer (using augmented matrix $\bar{R}$) is ${\bar{N}}^{2}$. If the covariance matrix R is used directly, the DOFs is $N^{2}$, although the two matrices have the same distinct elements. Referring to the two-level nested array, the optimal values $N_{1}$, $N_{2}$ and the corresponding DOFs of the adaptive beamformer are listed in Table 1.

It can be easily seen that $\bar{N}\geq N$ when N≥2, which means that larger DOFs for the adaptive beamformer can be obtained from the augmented matrix $\bar{R}$. Compared with the traditional method, which increases the DOFs by using more antenna elements, the NNFDA scheme utilizes the advantages of NLA and incorporates the reconstruction method to obtain the virtual matrix, having equal DOFs to the larger ULA. The larger DOFs are able to improve the output beamformer performances; namely, in terms of a better trade-off between the main lobe width and side lobes levels, and the higher SINR.

The normalized received beampattern $\bar{B}(r,\theta )$ and the output SINR are respectively given as

(28) $$\bar{B}(r,\theta )=\frac{{\left|{\bar{w}}^{H}{\bar{v}}_{1}(r,\theta )\right|}^{2}}{{\left|{\bar{w}}^{H}{\bar{v}}_{1}(r_{0},\theta_{0})\right|}^{2}}$$

(29) $$\mathrm{SINR}=\frac{{\sigma_{0}}^{2}{\left|{\bar{w}}^{H}{\bar{v}}_{1}(r_{0},\theta_{0})\right|}^{2}}{{\bar{w}}^{H}{\bar{\mathrm{Rw}}}^{H}-{\sigma_{0}}^{2}{\left|\left|{\bar{w}}^{H}{\bar{v}}_{1}(r_{0},\theta_{0})\right|\right|}^{2}}$$

## 3. Simulations and Discussions

In the numerical simulations, the reference carrier frequency is $f_{0}=1.6\;\mathrm{GHz}$, the frequency offset is set as Δf=5 kHz and the snapshot number is 500. The target is assumed to be located at a range $r_{0}=10\;\mathrm{km}$ and in the direction $\theta_{0}=30{}^{\circ}$. We consider the 8-element collocated NNFDA-MIMO Radar, the *i*th (*i* = 1, 2, 3, …, 8) element carrier frequency is the ith element of the set $\left[f_{0}+\Delta f,f_{0}+2\Delta f,f_{0}+3\Delta f,f_{0}+4\Delta f,f_{0}+5\Delta f,f_{0}+10\Delta f,f_{0}+15\Delta f,f_{0}+20\Delta f\right]$, and the set of the antennas’ positions is [d,2d,3d,4d,5d,10d,15d,20d]. From the 8-element collocated NNFDA-MIMO Radar’s received snapshots, we can obtain two resultant MVDR beamformings with different DOFs. One is obtained by directly using the covariance matrix while the other one utilizes the augmented matrix. In order to distinguish and compare the two MVDR beamforming results more clearly, we name the one that directly uses the covariance matrix as NNFDA direct, and the other one is NNFDA augmented. A better performance—for example, a better suppression of strong interference and a higher energy concentration—can be achieved with a greater number of DOFs [18,20]. To verify this, this paper introduces another two conventional FDA-MIMO Radars, the 8-element FDA-MIMO Radar and 20-element FDA-MIMO Radar, to carry out the simulations and comparisons. The noise is assumed to be White Gaussian Noise, and the SNR is equal to 0dB. In addition, the interferences, as described in (6), impinge the array from directions {20°,40°}. For each interference, its interference-to-noise ratio (INR) is 10dB. The four DOFs and number of distinct elements are provided in Table 2.

**Figure 4:**
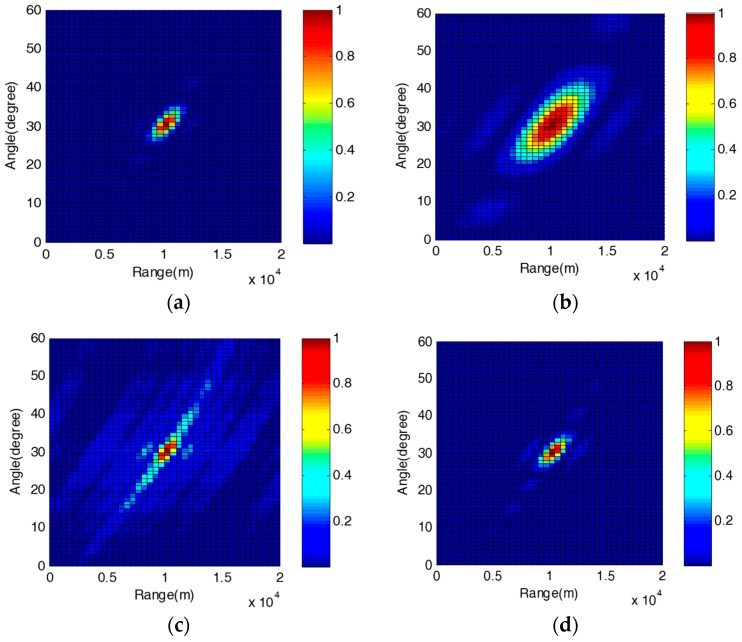
Normalized beampatterns of FDA-MIMO Radar and NNFDA-MIMO Radar: (**a**) 20-element FDA-MIMO Radar; (**b**) 8-element FDA-MIMO Radar; (**c**) 8-element NNFDA-MIMO Radar direct; (**d**) 8-element NNFDA-MIMO Radar augmented.

Figure 4a–d shows the normalized beampatterns of the Radars listed in Table 2. It can be found that the four normalized beampatterns’ peaks appear at the same point (10 km, 30°), which is the desired location of the target indeed. Hence, the effectiveness of the MVDR beamforming, for the FDA-MIMO Radar and NNFDA-MIMO Radar, can be verified. For the conventional FDA-MIMO Radar, a greater number of DOFs always means a sharper beampattern, but depends on more elements. Comparing Figure 4a,b, we find that the normalized beampattern of 20-element FDA-MIMO Radar (DOFs 400) obviously has a narrower main-lobe than the one of the 8-element FDA-MIMO Radar (DOFs 64). However, for the NNFDA-MIMO Radar, we can utilize the MVDR beamforming based on the augmented matrix to increase the DOFs, instead of only relying on a larger array. Figure 4c,d provides the resultant beampatterns of the 8-element NNFDA direct and 8-element NNFDA augmented. They have approximately the same main lobe width, but the latter possesses obviously lower side lobe levels, due to the increased DOFs (from 64 to 400). Figure 4c,d verifies the effectiveness and necessity of the augmented matrix, introduced in Section 3. Furthermore, although the numbers of elements in Figure 4b,d are the same, the normalized beampattern of 8-element NNFDA augmented is significantly better than the one of the 8-element FDA in terms of main lobe width and side lobe depth. The NNFDA-MIMO Radar can improve normalized beampattern with the same number of elements.

**Figure 5:**
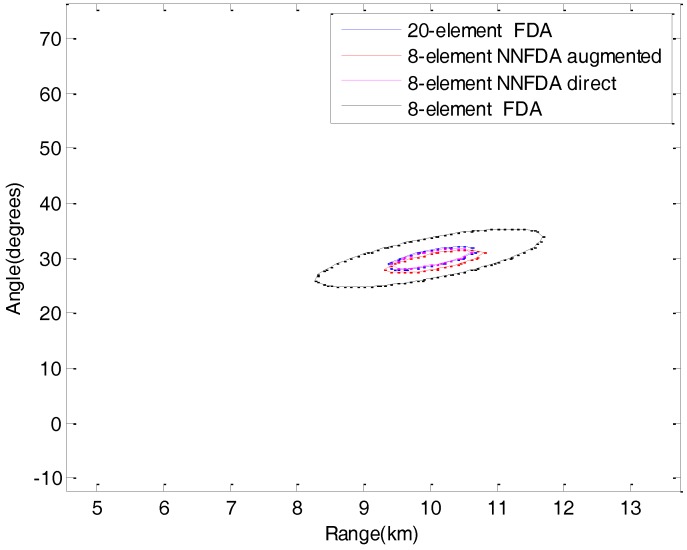
−3dB sectional areas of the normalized beampatterns.

In order to visually display the normalized beampatterns’ main-lobe width, Figure 5 plots the −3dB sectional areas which can effectively reflect the main lobes’ energy concentration performance. In Figure 5, the 20-element FDA-MIMO Radar, the 8-element NNFDA-MIMO Radar augmented and the 8-element NNFDA-MIMO Radar direct have nearly the same −3dB sectional area of beampattern, which is significantly narrower than the one of the 8-element FDA-MIMO Radar. Because those three have the same maximum aperture of equivalent antenna in theory. Through Figure 5, it can be concluded that the proposed FDA-MIMO Radar scheme can reduce the element number while maintaining the approximate normalized beampatterns’ main lobe width.

**Figure 6:**
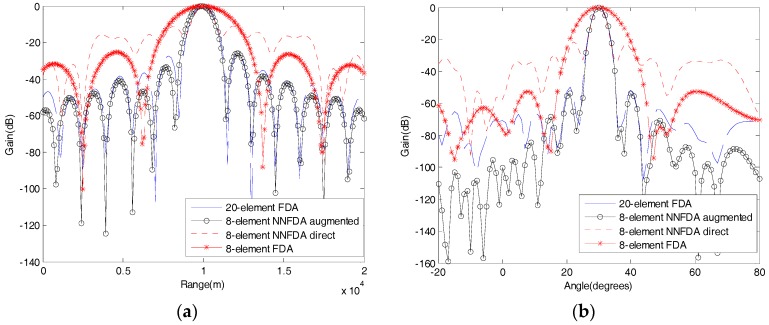
Ranges and angle scanning of the normalized beampatterns: (**a**) range scanning at the desired direction ($\theta_{0}=30{}^{\circ}$); (**b**) angle scanning at the desired range ($r_{0}=10\;\mathrm{km}$).

The results of the range scanning at the desired direction ($\theta_{0}=30{}^{\circ}$) are provided in Figure 6a. Among the four corresponding results, there are three (20-element FDA, 8-element NNFDA augmented, 8-element NNFDA direct) possessing nearly the same main lobe beam width, because their covariance matrices or augmented matrix, used for the received beamforming, have the same number of distinct elements. However, due to their having equal DOFs, the side lobes of the 8-element NNFDA augmented and the 20-element FDA are nearly identical to one another, but are much lower than those of 8-element NNFDA direct. A similar situation appears in Figure 6b, which shows the results of the angle scanning at the desired range ($r_{0}=10\;\mathrm{km}$). Generally, the resolution is defined as the width of the −3dB range in the normalized pattern, so we can assume that the received beampatterns of 20-element FDA and 8-element NNFDA augmented have a similar angle resolution and range resolution.

**Figure 7:**
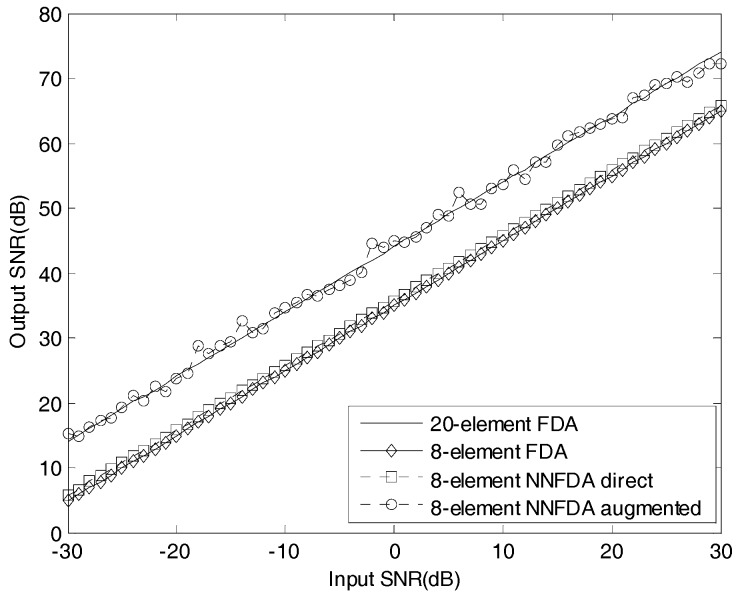
Output SINR versus input SINR.

The output SINR versus the input SINR for the described MVDR beamformers are plotted in Figure 7, where the input SINR is changed from −30dB to 30dB while the other parameters are the same as those in Figure 4. Through Figure 7, it can be concluded that in the aspect of robustness against the noise and interference, the 8-element NNFDA augmented beamformer is quite same to the 20-element FDA beamformer, but better than the 8-element NNFDA direct beamformer and the 8-element FDA beamformer.

## 4. Conclusions

In order to obtain larger DOFs, this paper researches the signal model of the FDA-MIMO Radar with nested frequency offset and nested array, then proposes an improved adaptive beamforming method that can provide a better beamforming performance, including narrower main lobe width, lower side lobes, and a higher output SINR, as shown in the simulations. Furthermore, the proposed method provides a novel working mode for the existing FDA-MIMO Radar. Such a mode can reduce hardware expense, as the process of the augmented matrix is not complex, creating a new array by selecting fewer elements to support a fully functional radar, rather than letting the whole array work as the traditional mode does. More importantly, the corresponding resultant received beampatterns obtained by the two different modes have the nearly same output performance.

## Figures and Tables

**Table 1:** DOFs of the adaptive beamformer.

N	Optimal $N_{1}$, $N_{2}$	R (DOFS)	$\bar{R}$ (DOFS)
even	$N_{1}=N_{2}=\frac{1}{2}N$	$N^{2}$	${\left(\frac{N^{2}}{4}+\frac{N}{2}\right)}^{2}$
odd	$N_{1}=\frac{N-1}{2},\;N_{2}=\frac{N+1}{2}$	$N^{2}$	${\left(\frac{N^{2}+1}{4}+\frac{N}{2}\right)}^{2}$

**Table 2:** The four DOFs and numbers of distinct elements.

	DOFs	Number Distinct Elements
20-element FDA-MIMO Radar	400	1521
8-element FDA-MIMO Radar	64	225
8-element NNFDA-MIMO direct	64	1521
8-element NNFDA-MIMO augmented	400	1521

## Abbreviations

FDA-MIMO	frequency diversity array multiple-input-multiple-output
NNFDA-MIMO	nested frequency offset and nested array FDA-MIMO
ULA	uniform linear array
DOFs	degrees of freedom
SINR	Signal to interference noise ratio
MRA	minimum redundancy array
MVDR	minimum variance distortionless response
